# Precise Characterisation of Molecular Orientation in a Single Crystal Field-Effect Transistor Using Polarised Raman Spectroscopy

**DOI:** 10.1038/srep33057

**Published:** 2016-09-13

**Authors:** Sebastian Wood, Grigorios-Panagiotis Rigas, Alina Zoladek-Lemanczyk, James C. Blakesley, Stamatis Georgakopoulos, Marta Mas-Torrent, Maxim Shkunov, Fernando A. Castro

**Affiliations:** 1 National Physical Laboratory, Hampton Road, Teddington, TW11 0LW, United Kingdom; 2 Advanced Technology Institute, University of Surrey, Guildford, GU2 7XH, United Kingdom; 3 Institut de Ciencia de Materials de Barcelona (ICMAB-CSIC), Campus UAB, 08193 Cerdanyola, Spain

## Abstract

Charge transport in organic semiconductors is strongly dependent on the molecular orientation and packing, such that manipulation of this molecular packing is a proven technique for enhancing the charge mobility in organic transistors. However, quantitative measurements of molecular orientation in micrometre-scale structures are experimentally challenging. Several research groups have suggested polarised Raman spectroscopy as a suitable technique for these measurements and have been able to partially characterise molecular orientations using one or two orientation parameters. Here we demonstrate a new approach that allows quantitative measurements of molecular orientations in terms of three parameters, offering the complete characterisation of a three-dimensional orientation. We apply this new method to organic semiconductor molecules in a single crystal field-effect transistor in order to correlate the measured orientation with charge carrier mobility measurements. This approach offers the opportunity for micrometre resolution (diffraction limited) spatial mapping of molecular orientation using bench-top apparatus, enabling a rational approach towards controlling this orientation to achieve optimum device performance.

Organic semiconductors have attracted interest as a promising alternative to traditional inorganic semiconductors for low cost, light-weight, flexible electronic devices. Their use in various applications has been demonstrated, including light-emitting diodes, photovoltaics, biosensors, and field-effect transistors^1^,^2^,^3^,^4^. The continued development of new organic semiconducting materials, processing techniques, and device architectures demands novel characterisation techniques, in particular, non-destructive, non-invasive measurements that can be deployed in an industrial context.

In this study, we consider specifically the measurement of molecular orientation, which has a profound impact on charge transport within an organic molecular crystal and determines the charge carrier mobility in an electronic device^5^. Multiple studies have shown that tuning the molecular orientation and packing motif using processing conditions, surface interactions, or chemical substitutions can have a profound impact on the resulting charge carrier mobility^6^,^7^,^8^,^9^,^10^,^11^. Several different techniques have been explored for molecular orientation measurements in thin film organic semiconductors, the most established of which is X-ray diffraction. This has been demonstrated for a number of thin film organic semiconductors, however, a high powered X-ray beam line is usually required to obtain a measurable signal, which limits the spatial resolution for *in situ* studies^6^,^9^,^12^,^13^. For these reasons, an optical spectroscopic approach is attractive as a more practical technique with micrometre spatial resolution (diffraction limited). Here we demonstrate polarised Raman spectroscopy as a technique for elucidating the orientation of molecules within a single crystal organic field-effect transistor.

The theoretical basis of polarised Raman spectroscopy and its relationship with molecular orientation are well-known, and helpfully reviewed by Tanaka *et al*.^14^,^15^. In contrast, the experimental realisation of polarised Raman spectroscopy as a probe of molecular orientation in organic semiconductors has proven challenging^16^,^17^,^18^,^19^,^20^,^21^,^22^. Published reports have typically focused on pentacene and the soluble derivative 6,13-bis(triisopropylsilylethynyl)pentacene (TIPS pentacene), which readily provide crystalline samples with good charge transport properties. Notably, Mino *et al*. were able to use polarised Raman spectroscopy to characterise the orientation of pentacene molecules in terms of two orientation angles, whereas James *et al*. made a qualitative comparison based on a single orientation angle^17^,^18^. However, to fully describe the orientation of a molecule such as pentacene requires three parameters, hence these previous results provide a locus of possible solutions rather than a specific measurement of orientation. Here we address and overcome several experimental limitations in order to demonstrate the characterisation of molecular orientation of TIPS pentacene in a single crystal sample in terms of three Euler coordinates. The measured orientation is in agreement with angle-dependent transistor mobility measurements, and other reports, but suggests a slight difference in interfacial molecular orientation with respect to previous studies based on X-ray diffraction of larger crystals.

## Results and Discussion

**Figure 1:**
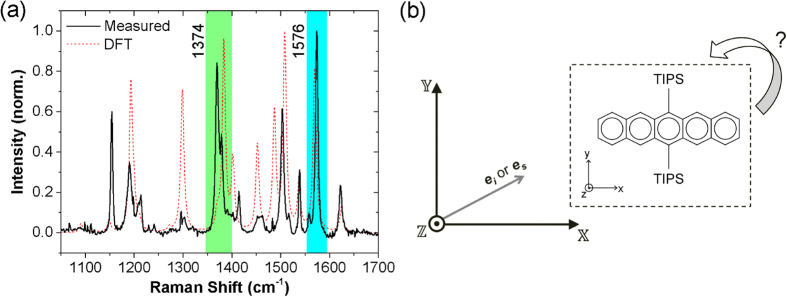
Raman spectrum of TIPS pentacene and illustration of reference frames. (**a**) Comparison of measured Raman spectrum of TIPS pentacene sample with DFT calculated spectrum showing modes in the range 1050–1700 cm^−1^ measured using 785 nm excitation. Highlighted peaks at 1374 cm^−1^ (green), and 1576 cm^−1^ (blue) are employed to probe the molecular orientation. (**b**) Diagram showing incident and sampled polarisation vectors (***e***_***i***_ and ***e***_***s***_) within the laboratory frame axes: 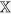, 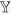 and 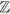. The TIPS pentacene molecule occupies the molecular frame with axes *x*, *y* and *z*, which has an unknown orientation within the laboratory frame.

In the general case, the intensity of polarised Raman scattering from a molecule for a particular vibrational mode, *j*, is given by:


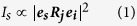


where *I*_*s*_ is the intensity of Raman scattering, ***R***_***j***_ is the Raman tensor of mode *j* for the molecule, ***e***_***i***_ is the electric field vector of the light incident on the molecule (controlled by a polariser), such that the product ***R***_***j***_***e***_***i***_ represents the electric field vector of the Raman scattered light, and ***e***_***s***_ is the electric field vector sampled by the instrument (controlled by an analyser). Figure 1(a) shows a typical Raman spectrum of TIPS pentacene, measured with a 785 nm excitation laser. The two strongest Raman peaks are measured at 1374 cm^−1^ and 1576 cm^−1^, corresponding to collective stretching modes of the acene core of the pentacene molecule, and the relative intensities of these modes show a strong dependence on the molecular orientation arising from the different forms of the Raman tensors for these modes^14^,^18^. The pentacene molecule (without TIPS groups) belongs to the D_2h_ point group and the corresponding peaks at 1371 cm^−1^ and 1596 cm^−1^ are assigned to A_g_ and B_3g_ modes, respectively^17^. The Raman tensors for these modes in the molecular frame, with the molecular orientation equivalent to that of the TIPS pentacene in Fig. 1(b), have the general forms:


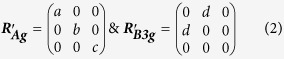


where *a*, *b*, *c*, and *d* are unknown values. Note that the Raman tensor for the B_3g_ mode differs from the usual form for a B_3g_ tensor due to the non-standard molecular orientation used here to facilitate comparison with TIPS pentacene. The addition of TIPS-groups breaks the symmetry of the pentacene molecule such that we expect the Raman tensors to adopt more complex forms. We therefore use density functional theory (DFT) calculations on the TIPS pentacene molecule to optimise the molecular geometry and simulate the Raman scattering (see Experimental Methods for details). The calculated spectrum is compared with the measured case in Fig. 1(a) and the clear correspondence between the measured 1374 cm^−1^ and 1576 cm^−1^ peaks and strong simulated modes enables us to match the measured modes with calculated Raman tensors, whose normalised values are:


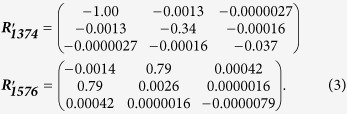


In fact, several elements in these normalised Raman tensors for TIPS pentacene are much less than unity, such that the overall form is similar to the theoretical tensors for pentacene. This is in line with the expectation that the Raman activity of TIPS pentacene is dominated by the conjugated pentacene core, and that the solubilising TIPS groups have little impact on the molecular optoelectronic properties. It is important to recognise that these Raman tensors are valid only in the non-resonant regime and so 785 nm excitation is used for experimental measurements since the sample does not absorb light at this wavelength.

Figure 1(b) shows the molecular structure of TIPS pentacene and its orientation within the molecular frame (*x*, *y*, *z* axes). The Raman tensors (***R***′) are also calculated in this molecular frame. The laboratory frame (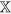, 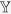, 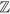 axes) is defined by the orientation of the instrument and the sample also lies in the laboratory frame. There are multiple ways to describe the orientation of a molecule and no clear consensus in the literature, so we adopt the system of extrinsic Euler coordinates, which is clearly set out by Roy *et al*. where the order of operations is: rotation by *ψ* about 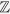; followed by rotation by *θ* around 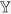; followed by rotation by *φ* around 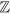
23. This gives a direction cosine matrix, ***D***:


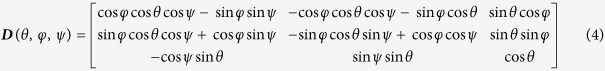


such that the Raman tensor, ***R***′_***j***_ and spatial coordinates, ***r***′, defined in the molecular frame can be rotated within the laboratory frame to give ***R***_***j***_, and ***r***.


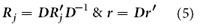


The objective of the measurement is to elucidate the rotation that must be applied to the molecular frame in order to bring the simulated molecule into the same orientation as the real molecules in the laboratory frame. This is achieved by measuring the polarisation dependence of the Raman scattering intensity. To do this, we vary the polarisation direction of the excitation laser, ***e***_***i***_ using a half-wave plate to rotate the linear polarisation vector through the angle, *θ*_*P*_. In addition, we use an analyser to sample different polarisation components of the scattered light, where the sampled polarisation,***e***_***s***_, is controlled by the orientation angle of the analyser, *θ*_*A*_. Experimentally, there are multiple components in the optical path with polarisation dependent characteristics (the Rayleigh scattering filter, in particular) resulting in a difference between the linear polarisation direction of the laser beam after the half-wave plate and when it reaches the sample. The polarisation of the scattered light is similarly affected. These effects can be adequately accommodated by introducing the correction factors, *k* and *m*, into ***e***_***i***_ and ***e***_***s***_, which makes the reasonable assumptions that these instrumental factors are symmetric with respect to the 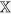 and 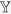 axes and have no appreciable impact in the 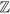 direction^24^:


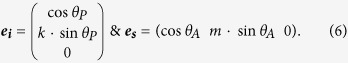


**Figure 2:**
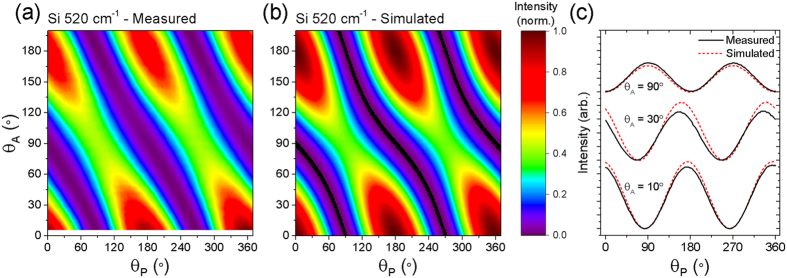
Polarised Raman spectroscopy results for silicon reference sample. Comparison of (**a**) measured, and (**b**) simulated plots of the 520 cm^−1^ Raman normalised peak intensity for the silicon reference sample plotted against the polariser angle (*θ*_*P*_) and analyser angle (*θ*_*A*_). (**c**) Compares profiles through the measured and simulated data for *θ*_*A*_ = 10, 30, 90°.

In order to obtain values for *k* and *m*, we use a silicon wafer reference sample and measure the integrated intensity of the 520 cm^−1^ peak as a function of *θ*_*A*_ and *θ*_*P*_, the results of which are plotted in Fig. 2(a). The minimum intensity value is subtracted as a first-order correction for partial depolarisation of the incident and scattered light arising in the instrument, and the resulting values are normalised. A more formal accommodation of the partial depolarisation would require an alternative formulation of the system using Mueller calculus, however this would greatly increase the complexity and we find that a good fit between experimental and simulated results can be achieved using our simpler methods.

For the silicon reference sample, the 520 cm^−1^ peak comprises three degenerate modes with the tensors:


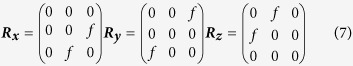


in the system with crystal coordinates: *x* = [100], *y* = [010], *z* = [001], and where *f* is some unknown value^25^. Substituting these tensors into Equation (1), and using the relationships in Equation (6) we are able to simulate the resulting polarised Raman scattering intensity map corresponding with the experimental measurement. Since the primary flat of the silicon reference sample is parallel to the <110> direction, a rotation of 45° around the *z*-axis is required to bring the tensors in Equation (7) into the same orientation as the reference sample. Taking initial values of *k *= *m* = 1, the least squares difference between the measured and simulated values for a full matrix of *θ*_*A*_ and *θ*_*P*_ values over the measured range is minimised using a MATLAB script to find that the best fit is achieved when *k* = 0.829 and *m* = 0.747. The resulting simulated data are shown in Fig. 2(b).

The fitting of the experimental data with the simulated results for the silicon reference sample demonstrates that the model is able to reproduce the main features of the experiment, with the maxima and minima close to the measured *θ*_*A*_ and *θ*_*P*_ values. However, we note that there are some minor discrepancies, particularly in the relative intensities, which are illustrated by the comparison of profiles in Fig. 2(c) for several values of *θ*_*A*_. Considering silicon to be a good reference material, we attribute the imperfect fitting to the incomplete representation of instrument properties in the model, specifically, the depolarisation effects mentioned above. The impact of these factors on the reliability of the fitting is minimised by collecting data over an extended range of *θ*_*A*_ and *θ*_*P*_ values (in principle, 0 < *θ*_*A*_ < 90° and 0 < *θ*_*P*_ < 180° would give a complete data set).

Using the values for *k* and *m* evaluated from the silicon reference sample, we apply the technique to a single crystal of TIPS pentacene, where the molecular orientation is unknown. An image of the sample is given in Fig. 4(a), showing the TIPS pentacene crystal (roughly 150 μm × 80 μm laterally with a thickness of 220 nm, estimated using atomic force microscopy) deposited onto a radial array of gold electrodes. These electrodes enable us to make charge transport measurements through the sample using different pairs of electrodes as source and drain to measure transistor mobilities for different orientations of the transistor channel.

**Figure 3:**
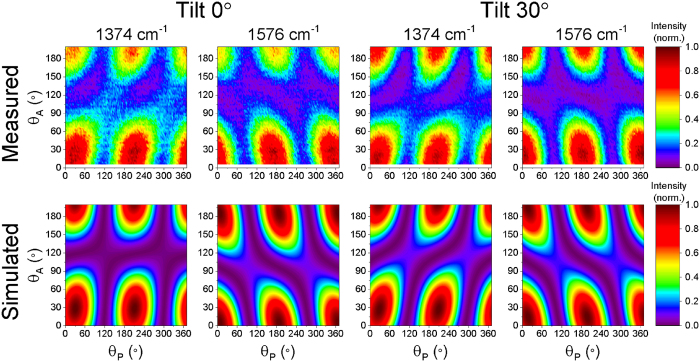
Polarised Raman spectroscopy results for TIPS pentacene sample. Comparison of measured (top) polarised Raman data with the best fitting simulated results (bottom). The intensities of the 1374 cm^−1^ and 1576 cm^−1^ modes measured with tilts of both 0° and 30° around the 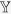-axis are considered. In each plot the normalised intensity of the Raman peak is plotted against the polariser angle (*θ*_*P*_) and analyser angle (*θ*_*A*_).

In this work we assume an idealised single crystal of TIPS pentacene where all the molecules share a single orientation: in reality we expect some degree of disorder resulting in a distribution of molecular orientations within the sample. In order to obtain sufficient polarised Raman data to assign the measured results to a single molecular orientation, we extract the integrated intensities of both the 1374 cm^−1^ and 1576 cm^−1^ modes. The measurement is also performed with the sample in two different orientations by applying tilt angles to the sample of 0° and 30° around the 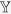-axis. This is necessary because the results from a single sample orientation will typically yield two indistinguishable solutions, which are reflections of each other in the 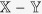 plane, so by tilting the sample out of this plane these two cases can be distinguished. The intensities of the 1374 cm^−1^ and 1576 cm^−1^ peaks for both tilt angles are shown in Fig. 3(a), with each of the four plots normalised to its own maximum.

**Figure 4:**
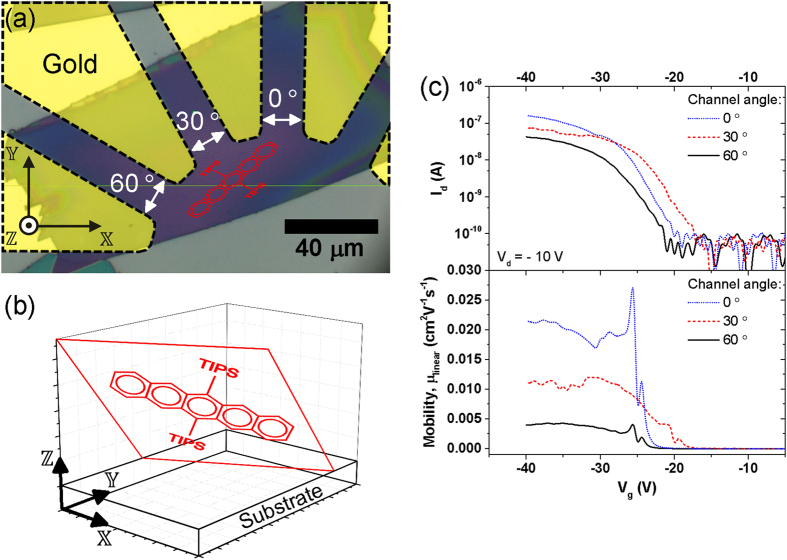
Orientation dependence of charge carrier mobility in TIPS pentacene sample. (**a**) Microscope image of sample showing a TIPS pentacene crystal deposited on top of an array of radial gold electrodes (outlined and highlighted) to form transistor channels with different angles (0, 30, and 60°) between the charge transport direction (white arrows) and 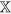-axis. The orientation of the TIPS pentacene molecule is shown in red, projected on to the 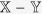 plane. (**b**) Plot showing the TIPS pentacene molecular structure in its measured orientation with respect to the substrate in the laboratory frame (plane of the acene backbone also shown for clarity).(**c**) Comparison of measured transfer characteristics (top) and estimated hole mobilities assuming the linear operation regime (bottom) for transistor channels at 0, 30, and 60°.

As before, a simulation of the polarised Raman scattering measurement is carried out, this time using the calculated Raman tensors given in Equation (3) and substituting the rotated Raman tensors, ***R***_***j***_ from Equation (5) into Equation (1). Least-squares fitting is used to find the set of Euler coordinates, *ψ*, *θ* and *φ*, which rotate the Raman tensors to give the best fit of the simulation to the experimental data. Figure 3 compares the best fitting simulation results with the measured data. As for the silicon reference sample, the simulation is able to reproduce the main features of the experimental data, but here the imperfect fitting represents a combination of both instrumental factors such as depolarisation, and non-ideal sample properties, such as orientational disorder. The robustness of the result is confirmed by repeating the fitting using a series of 196 combinations of uniformly spaced initial values for the Euler coordinates, and we find that all the best fit values correspond to equivalent molecular orientations (within tolerances of < 1° for each coordinate). The values: *ψ* = 68.3°, *θ* = 118.5°, *φ* = −124.6° are found to describe the rotation of the TIPS pentacene molecule from the molecular frame into the same orientation as the sample molecules in the laboratory frame. Figure 4(b) represents this molecular orientation by transforming the spatial coordinates of the TIPS pentacene atoms, ***r***′, (excluding alkyl groups and hydrogen atoms) into the laboratory frame, ***r***, using equation (5).

Comparing the molecular orientation derived from this method with other published reports reveals a broad agreement. In particular, we note that our solution shows the TIPS groups tilted closest to the substrate, and the shape of the crystal as a whole, shown in Fig. 4(a), is consistent with this orientation too^26^,^27^. Importantly, X-ray diffraction (XRD) studies of similar samples typically show the long axis of the pentacene lying near to parallel with the substrate plane, whereas our result shows a small but significant tilt^13^,^28^,^29^. It was not possible to measure XRD data for these single crystal samples due to their size and sensitivity to radiation damage, though this would have enabled a valuable comparison. Rather, this difficulty highlights the need for alternative techniques, such as the one developed here, which can probe molecular orientations non-destructively in small, sensitive samples. XRD studies on larger samples have shown that the molecular packing of various acene derivatives depends on crystallite size and growth conditions, and so it is reasonable to conclude that the result presented here reveals a real phenomenon^6^,^22^,^30^.

The charge transport in TIPS pentacene crystals is anisotropic since the charge transfer is mediated through overlapping π-orbitals of the acene core and the molecules adopt a ‘brick-wall’ packing motif^10^,^31^. We can therefore verify the molecular orientation, measured using polarised Raman spectroscopy, by comparing this with the angular dependent charge mobility using the transistor structure shown in Fig. 4(a). Three different transistor channels are formed in the sample with angles of 0, 30, and 60° between the charge transport direction and the 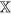-axis. We expect to measure the lowest charge mobility when the acene core is aligned parallel with the channel length and higher values when it is aligned at a more oblique angle. The limited number of devices available in this sample mean that quantitative conclusions cannot be drawn, but the transfer characteristics and mobility values shown in Fig. 4(c) show a significantly higher mobility for the device with a 0° channel angle, corresponding with an angle of ~40° between the channel orientation and the 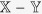 projection of the acene core, than for the 30° and 60° devices, where the acene is closer to parallel to the channel current.

## Conclusions

This study has demonstrated the use of polarised Raman spectroscopy as a probe for molecular orientation, taking the example of a TIPS pentacene single crystal transistor as a model system. Importantly, we have been able to express the solution quantitatively using Euler coordinates to fully describe the orientation of the molecule with respect to the substrate. This work opens the way for meaningful and unambiguous measurements of molecular orientation using a non-destructive, non-invasive, bench-top technique capable of offering diffraction-limited lateral resolution. Further refinement of this method might be achieved by incorporating additional factors into the simulation to account for instrumental depolarisation effects more explicitly, or with additional parameters to describe the orientational disorder present in the sample. In the example considered here, the molecular orientation of TIPS pentacene in the small single crystal is found to differ slightly from that reported for larger crystals. Subtle modifications to the bulk molecular packing motif in thin films have been found to have dramatic impacts on the charge transport properties of organic semiconductors, and so polarised Raman spectroscopy offers a particularly appropriate tool for probing molecular orientations in thin film or microstructured devices. Combining this technique with methods to control the molecular orientation provides a way to intelligently tune the fabrication conditions for optimised performance of organic molecular electronics.

## Experimental Methods

### Sample Preparation

A circular array of Au electrodes was patterned on top of a SiO_2_/Si substrate using conventional photolithography^32^. The substrate used was n++ Si with 200 nm thermally grown SiO_2_ (purchased from Si-Mat). S1813 positive photoresist was spin-coated at 4000 rpm, followed by baking at 100 °C for 1 min. The photoresist was patterned with a laser writer (Durham magneto-optics) at 405 nm with a dose of 150 mJ/cm^2^. The sample was developed with MF319 developer for 1 min and rinsed with deionised H_2_O. Subsequently the metal layers were evaporated; 5 nm of Cr (rate 0.3 Å/s) followed by 35 nm of Au (rate 0.5 Å/s). Lift-off was performed by sonicating in acetone and further cleaning by sonicating in HPLC grade acetone and isopropanol. For the single crystals, 2 mg of TIPS-pentacene, synthesised with previously described techniques^28^, was dissolved in 1 mL of toluene. The solution was spray-printed, using a previously reported technique^33^, on top of the electrodes forming single crystals. The electrode/organic and dielectric/organic interfaces were not modified using any self-assembled monolayer prior to the crystal deposition.

The lateral dimensions of the crystal were measured using optical microscopy, and the vertical thickness measured by atomic force microscopy using an MFP-3D (Asylum Research) instrument in tapping mode. Line profiles across the crystal edge were measured in three places giving values of 221, 220, and 210 nm as approximations for the crystal thickness.

### Polarised Raman Spectroscopy

Raman spectra were measured using a Horiba HR800 Evolution Labram Spectrometer. A 785 nm excitation laser was used with a half-wave plate polariser and an analyser for polarised measurements. The angular position of the polariser, *P*, is related to *θ*_*P*_ and the resulting orientation of the incident polarisation vector, ***e***_***i***_, using: 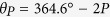, which accounts for the behaviour of the half-wave plate and also an offset from zero of the polariser position. Similarly, the position of the analyser, *A*, relates to *θ*_*A*_ and the orientation of the sampled polarisation vector, ***e***_***s***_, using: 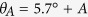. The sample is mounted on a ThorLabs PRM1/MZ8 rotating stage to control the sample tilt around the 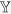-axis. Polarisation dependent Raman plots were acquired for all points in the range 0 < *P* < 190 in 1.5° steps, and 0 < *A* < 200 in 3° steps with a 1 s acquisition time at each point. The laser power at the sample was ~10 mW for TIPS pentacene samples and ~100 mW for the silicon reference. No change in the Raman spectrum of pentacene was observed after multiple excitations indicating that no significant sample degradation occurred during the measurement. A 50× long working distance objective was used throughout to accommodate the tilting of the sample up to 30° out of plane, the numerical aperture of this objective (0.45) is sufficiently low enough that the depolarisation effect introduced by focusing the laser beam is not expected to be problematic^14^.

### DFT Calculations

DFT calculations were carried out using the GAUSSIAN09 software package to optimise the geometry of the TIPS pentacene molecule and then perform frequency analysis^34^,^35^. The B3LYP hybrid functional and the 6–31G(d,p) basis set were used^36^,^37^,^38^,^39^. The alkyl groups were truncated to minimise the computational requirements, and Raman tensors were extracted using a script developed by the Schlegel group^40^.

### Device Characterisation

For the electrical characterisation, a Keithley 4200 semiconductor characterisation system was used. Each transistor channel was defined by the fixed distance between the electrodes (L = 20 μm) and the width, W, of the crystal that was crossing it (W(60° device) = 70 μm, W(30° device) = 40 μm, W(0° device) = 60 μm) - see Fig. 4(a). The field effect mobility of each device was extracted using the standard MOSFET equation in the linear regime: 
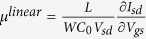
.

## Additional Information

**How to cite this article**: Wood, S. *et al*. Precise Characterisation of Molecular Orientation in a Single Crystal Field-Effect Transistor Using Polarised Raman Spectroscopy. *Sci. Rep*. **6**, 33057; doi: 10.1038/srep33057 (2016).
